# Motor Vehicle Crash and Hospital Charges in Front- and Rear-Seated Restrained and Unrestrained Adult Motor Vehicle Occupants

**DOI:** 10.3390/ijerph192013674

**Published:** 2022-10-21

**Authors:** Joyce C. Pressley, Emilia Pawlowski, Leah M. Hines, Sabana Bhatta, Michael J. Bauer

**Affiliations:** 1 Departments of Epidemiology and Health Policy and Management, Columbia University, New York, NY 10032, USA; 2 New York State Department of Health, Bureau of Occupational Health and Injury Prevention, Albany, NY 12237, USA

**Keywords:** restraint use, motor vehicle crash, injury severity, rear-seated adults, health care charges

## Abstract

There are reports that historically higher mortality observed for front- compared to rear-seated adult motor vehicle (MV) occupants has narrowed. Vast improvements have been made in strengthening laws and restraint use in front-, but not rear-seated occupants suggesting there may be value in expanding the science on rear-seat safety. Methods. A linked 2016–2017 hospital and MV crash data set, the Crash Outcomes Data Evaluation System (CODES), was used to compare characteristics of front-seated (*n* = 115,939) and rear-seated (*n* = 5729) adults aged 18 years and older involved in a MV crash in New York State (NYS). A primary enforced seat belt law existed for front-seated, but not rear-seated occupants. Statistical analysis employed SAS 9.4. Results. Compared to front-seated occupants, those rear-seated were more likely to be unrestrained (21.2% vs. 4.3%, *p* < 0.0001) and to have more moderate-to-severe injury/death (11.9% vs. 11.3%, *p* < 0.0001). Compared to restrained rear-seated occupants, unrestrained rear-seated occupants had higher moderate-to-severe injury/death (21.5% vs. 7.5%, *p* < 0.0001) and 4-fold higher hospitalization. More than 95% of ejections were unrestrained and had 7-fold higher medical charges. Unrestrained occupants’ hospital stays were longer, charges and societal financial costs higher. Conclusions. These findings extend the science of rear-seat safety in seriously injured rear-seated occupants, document increased medical charges and support the need to educate consumers and policy makers on the health and financial risks of adults riding unrestrained in the rear seat. The lack of restraint use in adult rear-seated motor vehicle occupants consumes scarce health care dollars for treatment of this serious, but largely preventable injury.

## 1. Introduction

Despite reports that restraint use is associated with lower mortality in both front- and rear-seated passengers, there remains a belief that beyond childhood, rear-seated passengers do not need to be restrained to travel safely [1,2,3,4,5,6,7,8]. Contrary to public opinion of little to no risk associated with being unbelted in the rear seat, there are reports that crash mortality for rear-seated occupants could be reduced by 55–75% by safety belt use [1,9]. Although it is likely that the restraint impact observed in fatal crashes is also applicable to non-fatally injured occupants, there is little information on the relative disparities in injury severity, discharge outcomes and associated health care costs for rear-seated compared to front-seated occupants [10,11,12]. Expanding this paucity of research on the full adult age span could support and catalyze policy makers to address gaps in adult rear seat safety belt laws and possibly nudge consumer behavior toward narrowing the currently existing sizeable disparities of higher front- than rear-seat restraint use [10].

Few states have all age seat belt laws for rear-seated passengers and even fewer have primary laws that allow enforcement of seat belt violations without requiring another violation as the reason for the stop [13]. During this study timeframe, New York State (NYS) had no law requiring rear-seat seat belt use for ages 16 years and older except for some exceptions when driven by a junior driver covered by graduated driver license laws [13].

Much of the public opinion regarding rear-seat safety is based on the historically lower mortality rates for rear-seated compared to front-seated occupants, particularly those involved in frontal crashes [14,15]. However, as restraint use approached 90% in front-seated, but not rear-seated occupants, and vehicle designs advanced to protect front-seated occupants with improved occupant cages and advanced airbags, there are reports that the safety advantage between front- and rear-seated occupants has narrowed [16,17,18,19].

Many current studies of rear-seated passengers focus on or include children making it difficult to assess the impact on adults and elderly occupants [20,21]. The majority of states have gaps in covered ages for restraint requirements for rear-seated passengers, which is hypothesized to be due, at least in part, to a lack of convincing studies that examine the full social, financial, and health impacts related to restraint use in adult rear-seated passengers.

This is the first study comparing crash outcomes and medical charges in adult front- and rear-seated restrained and unrestrained motor vehicle crash occupants using the linked Crash Outcomes Data Evaluation System (CODES) data. CODES is used to compare characteristics of front- and rear-seated adults involved in a motor vehicle crash in NYS, specifically examining: (1) injury outcomes, hospitalization length of stays, and discharge status; (2) vehicle and crash characteristics and (3) health care (hospitalizations and emergency department (ED) charges by restraint status in front- vs. rear-seating positions.

## 2. Materials and Methods

### 2.1. Data Source(s)

NYS CODES for calendar years 2016–2017 was used to examine characteristics between seating position and restraint status in this study. CODES uses a probabilistic linkage to match motor vehicle crash data from the NYS Department of Motor Vehicles (DMV) to the New York Statewide Planning and Research Cooperative System (SPARCS) which maintains hospitalization and emergency department data in NYS [20,21,22]. LinkSolv is the current commercial version of the linkage software originally funded by the National Highway Traffic Safety Administration [22,23,24]. LinkSolv has been widely used by the CODES network funded by the National Highway Traffic Safety Administration [22].

### 2.2. Study Population

The study population was identified from the original data set of all identified and linked crashes in NYS for 2016–2017. The population for this study was determined first by excluding infants, children and adolescents who have been previously reported [20,21]. Next, persons who were traveling on bicycles, foot, or in buses which may not have seat belts were excluded. Once adults aged 18 years and older who were occupants of a four-wheeled passenger vehicle were identified, passengers with missing restraint status and those who were recorded as cargo area passenger, riding/hanging on outside and other/unknown were excluded. The final study population included 121,668 adults aged 18 years or older who were occupants of a four-wheeled passenger vehicle involved in a motor vehicle crash in NYS in calendar years 2016 or 2017. Of these, 115,939 (95.29%) were front-seated and 5729 (4.71%) were rear-seated. Drivers were analyzed separately in passenger comparisons of front- vs. rear-seated passengers but included in analyses of front-seated occupants. Occupants of non-four-wheeled passenger vehicles, pedestrians and cyclists were excluded from analysis.

### 2.3. Exposure Variables

*Seating position.* Seating positions were classified as either front- (seating row 1) or rear-seated (seating rows 2 or higher). Drivers, front middle passengers, and front right passengers were categorized as front-seated occupants. Where data was available, seating positions were examined further by seating location within the row (1 and 2 or higher) and characterized as inboard (middle seating position) or outboard (right or left seated). Seating positions recorded as cargo area passenger, riding/hanging on outside and other/unknown were excluded.

*Restraint use.* Restraint use was dichotomized as restrained or not restrained.

### 2.4. Outcome Variables

*Charges.* Hospitalization and emergency department charges are reported in U.S. dollars for 2016–2017 rounded to the nearest dollar. Zero and inconsistent charges were set to missing and were not included calculations of mean, median or total charges. Total charges included hospitalization and emergency department charges, professional fees, tests, procedures incurred during the emergency department visit or hospitalization, but do not include follow-up, rehabilitation or out-of-hospital care charges [25].

*Injury severity.* Occupant injury severity was assessed using the KABCO score assessed at the crash scene. The KABCO score categorized injury as killed, suspected serious injury, suspected minor injury, possible injury, and no apparent injury [26].

### 2.5. Covariate Variable Definitions

#### 2.5.1. Occupant Characteristics

*Driver age*. Drivers were examined using the categories of ≤ 25, 25–44, 45–64, and 65 years and older.

*Passenger age.* Passengers were categorized as: ≤25, 25–44, 45–64, and 65 years and older.

*Sex/gender*. Sex/gender for drivers and passengers was classified as male, female or unknown/other.

#### 2.5.2. Vehicle Characteristics

*Vehicle body type*. Vehicle body types were characterized as car, light truck (sports utility vehicle), large truck and other.

*Vehicle year.* The model year of the vehicle was recorded and analyzed in the following categories: <1994, 1994–1997, 1998–2004, 2005–2008, 2009–2011, 2012 or newer, and unknown model year [27].

#### 2.5.3. Crash Characteristics

*Crash type.* Type of crash was categorized as a frontal, same-side, other side, or rear crash.

*Rollover.* Rollover was dichotomized as a yes/no variable according to whether or not the vehicle rolled over during the crash.

*Ejection.* The ejection status of an occupant resulting from a motor vehicle crash was categorized as not ejected, partially ejected, ejected or not reported/unknown.

*Airbag deployment.* Airbag deployment was dichotomized as a yes/no variable based on airbag deployed at the time of crash.

*Vehicle towed.* Whether or not a vehicle was towed after the crash was categorized as yes or no.

*Alcohol involvement*. Alcohol involved crash was dichotomized as a yes/no variable depending on whether the crash was characterized as alcohol involved.

*Speed involvement.* Speed involvement in the crash was dichotomized as a yes/no variable.

*Location.* The location of the motor vehicle crash was characterized as an urban or rural crash based on the Federal Highway Administration definition of a rural location as one with fewer than 5000 people and an urban location as greater than 5000 people [28].

*Evening crash.* An evening crash was defined as a crash occurring at 6 p.m. or later. A crash occurring on a school or work night was a crash occurring Sunday through Thursday, while a weekend crash was considered Friday or Saturday.

### 2.6. Hospitalization Length of Stay, Discharge and Insurance Payment Characteristics

*Disposition of admitted inpatients.* Patient disposition for hospitalizations was categorized as: (1) home, no care; (2) home, home health care; (3) other acute care facility; (4) skilled nursing facility; (5) died in the hospital; and (6) other/unknown patient disposition [25].

*Length of hospitalization.* Length of stay was measured in days for patients that were hospitalized.

*Source of payment*. Sources of payment for medical care included payment source by principal reimbursement payor for charges incurred in an emergency department and hospitalization acute care charges. Principal reimbursement, or the primary payor of emergency department and hospitalization charges from the motor vehicle crash admission, was categorized as: (1) private; (2) governmental source (Medicare, Medicaid, CHAMPUS/VA, and other governmental sources); (3) workman’s compensation; (4) self-pay/uninsured; (5) other payor; and (6) unknown payment source. The “other” category was quite heterogeneous [25].

### 2.7. Statistical Analyses

The 2016–2017 DMV and SPARCS data were standardized and linked using probabilistic record linkage. This technique utilizes Bayesian linkage which accounts for a lack of strong personal identifiers and is described elsewhere [22]. Linkages use Link Solv 8.3 software (Strategic Matching, Inc., Morrisonville, NY, USA) and are conducted at the NYS Department of Health in Albany, NY, by the Bureau of Occupational Health and Injury Prevention. Using data pooled across three linkage iterations, Strategic Matching, Inc. documented a 99.96% sensitivity and an 82.98% specificity for data linkages. This descriptive study used this linked data, to assess the bivariate relationships between the exposure (restraint use) and categorical outcomes (injury severity/mortality) by front- vs. rear-seating positions using Chi Square for categorical variables. In addition, subgroup comparisons using Chi Square were made for those seated in the rear seat for restrained vs. unrestrained and similarly among those front-seated by restraint use. Continuous variables are reported with mean and standard deviation. Statistical analyses of proportional differences used the Chi-Square test. All analyses accounted for the imputed structure of the data for this descriptive study. Statistical analyses were conducted using SAS Enterprise version 9.4, SAS Institute Inc., Cary, NC, USA. A *p*-value ≤ 0.05 was considered statistically significant [29,30].

## 3. Results

### 3.1. Population Characteristics

The study population consisted of 121,668 occupants aged 18 years and older. Of these, 115,939 (95.3%) were front-seated occupants and 5729 (4.7%) were rear-seated occupants (Table 1). Of the 115,939 front-seated occupants, 96,885 (83.6%) were drivers and 19,054 (16.4%) were front-seated passengers.

***Age.*** Adult rear-seated passengers were younger than front-seated drivers or passengers (Table 1). Nearly one-third (30.8%) of rear-seated passengers were under age 25 and 56.1% were under age 35 years (not shown in table). In contrast, adults aged 65 and older were more likely to be front-seated passengers compared to rear-seated passengers (12.7% vs. 8.6%, *p* < 0.0001) (Table 1).

***Sex/gender.*** Although slightly more than one-half (53.3%) of drivers were male, males comprised nearly three-fourths (72.3%) of unrestrained drivers. This difference was not observed for front-seated passengers, where females comprised approximately half of unrestrained front-seated passengers (Table 1).

*Restraint use.* Although the magnitude of differences in restraint use varied by age, rear-seat restraint use was lower than that of front-seated occupants in all age groups. Absolute differences in front-to rear-seat restraint use were highest in 25–34 year olds (20.2%) and lowest for passengers aged 75–84 years where the rear-seat restraint use differential narrowed to 10.1% (subgroup not shown in table). Compared to front-seated occupants, those rear-seated were more likely to be unrestrained (21.2% vs. 4.3%, *p* < 0.0001) and to have more moderate-to-severe injury/death (11.9% vs. 11.3%, *p* < 0.0001) (Table 1).

**Figure 1:**
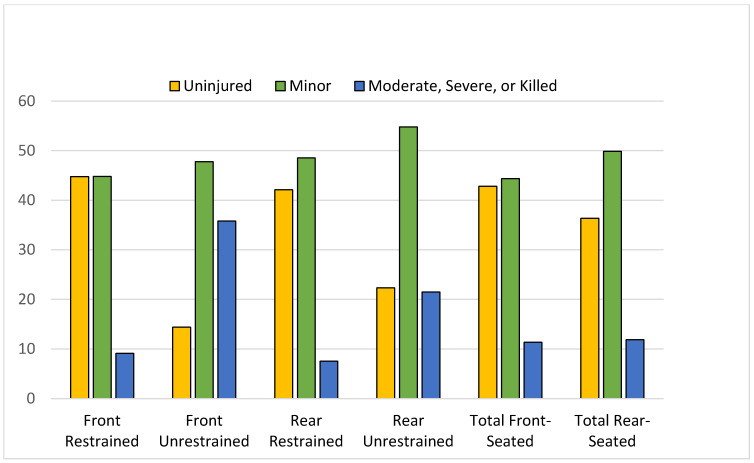
Injury severity is shown as the percent of occupants who sustained no injury (yellow), minor injury (green) and moderate or severe injury or died (blue) by seating position and restraint status with total front seated and total rear seated.

*Injury*. Rear-seated occupants were more likely to sustain injury than front-seated occupants (63.7% vs. 57.2%, *p* < 0.001), but this was driven largely by the higher proportion of rear-seated passengers who were unrestrained at the time of crash (Table 1). Rear-seated occupant injury was higher than for front-seated occupants for both minor injury and moderate-to-severe injury categories (Figure 1). Fewer rear-seated occupants escaped injury compared to front-seated occupants (14.4% vs. 22.3%, *p* < 0.0001). Among the restrained, rear-seated occupants sustained fewer moderate-to-severe injuries than front-seated occupants (7.5% vs. 9.1%, *p* < 0.0001).

Unrestrained occupants were more likely to be injured and to be injured more severely than those who were belted. Restraint use differentials between front- and rear-seated occupants varied by age, with lower front- to rear-seat restraint disparities observed in older ages (Table 1). Unrestrained occupants were 4 times more likely to be injured severely than those who were restrained. Among the rear-seated, approximately one-fifth (21.5%) of the unrestrained were moderately-to-severely injured or died, compared to 7.5% of restrained rear-seated passengers (*p* < 0.0001). Ninety percent (9 of 10) of rear-seated deaths were unrestrained.

### 3.2. Vehicle Characteristics

*Vehicle type*. Rear-seated occupants were less likely to be restrained than front-seated occupants, but this varied by vehicle type. In cars, rear-seated occupants were 10 times more likely to travel unrestrained than front-seated occupants. Among those in cars, one quarter of rear-seated occupants were unrestrained compared to fewer than 3% of front-seated occupants (*p* < 0.0001). These differences were consistent across all vehicle types, but differences were largest in cars (Table 2).

*Vehicle age.* Restraint status was similar between front- and rear-seating positions in older vehicles with model years before 1994 with about one-fifth in each seating position traveling unrestrained. This proportion of being unrestrained dropped significantly in newer vehicles for front-seated passengers, but not for rear-seated passengers where at least one quarter to one fifth continued to ride unrestrained (Table 2).

### 3.3. Crash Characteristics

*Ejection.* Approximately 95% of complete ejections in both front- and rear-seated occupants were unrestrained. The majority of partial ejections were unrestrained in both front-seated (71.6%) and rear-seated (60.0%) occupants (Table 2).

*Speed involvement*. Although small, but significant differences were noted in regard to rear-seated occupants being more likely to be involved in a speed-related crash than front-seated ones (7.9% vs. 7.2%, *p* = 0.002), there were stark differences in restraint status between rear-seated vs. front-seated occupants. Rear-seated occupants involved in a speed-related crash were 4.5 times more likely to be unrestrained than front-seated occupants (33.1% vs. 7.4%, *p* < 0.0001) (Table 2).

*Drivers in alcohol-involved crashes*. There were more than 2000 drivers who were documented to be in alcohol-involved crashes during the two-year timeframe of this study. Alcohol-involved drivers were 4.4 times more likely to be unrestrained. More than one-fifth of drivers who were restrained (27.3%) and unrestrained (28.0%) were missing data on whether the crash was alcohol involved.

*Location.* In rural areas, rear-seated occupants were 4.6 times more likely to be unrestrained than front-seated occupants (Table 2). However, traveling unrestrained was higher in rural compared to urban crashes in both front-seated (4.8% vs. 3.4%, *p* < 0.0001) and rear-seated occupants (21.8% vs. 19.0%, *p* < 0.0001, subgroup statistics not shown in table).

### 3.4. Resource Use and Medical Charges

*Length of hospitalization.* Length of stay tended to be slightly higher in rear-seated than front-seated passengers (Table 1). This difference was driven by the substantially higher proportion of rear-seated passengers who were unrestrained. Among the restrained, rear-seated passenger, mean length of stay was one day shorter than for front-seated passengers. Among rear-seated passengers, length of stay was nearly double in unrestrained compared to restrained occupants (Table 1).

Compared to restrained rear-seated occupants, unrestrained rear-seated occupants had higher moderate-to-severe injury/death (21.5% vs. 7.5%, *p* < 0.0001, subgroup statistics not shown in table) and 4-fold higher hospitalization. Hospitalization stays were longer, hospitalization charges higher and societal financial costs higher as the unrestrained were more frequently uninsured/self-insured/government-insured.

**Figure 2:**
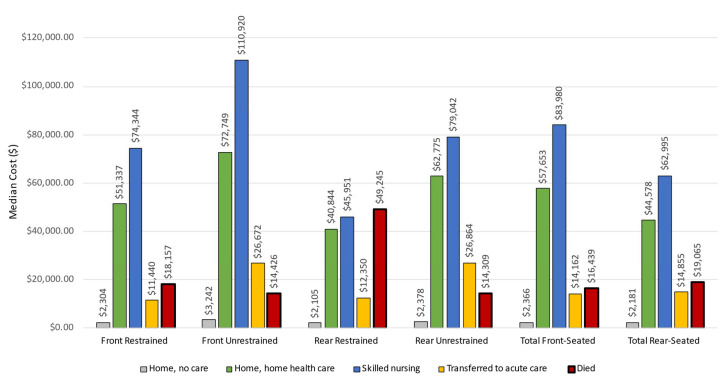
This figure shows hospital charges by front and rear seating positions and by restraint status and hospital discharge status: Home, no care (shown in gray), home with home health care (green), skilled nursing home (blue), transferred to another acute care facility (yellow) and died (red).

*Medical charges in front-vs. rear-seated occupants.* Total median hospital charges were only slightly higher in front- than in rear-seated occupants, but this differed by restraint status (Figure 2, Table S1, Supplementary Materials). Both front- and rear-seated unrestrained occupants were more likely to require additional care after discharge or transfer to another acute care hospital than similarly seated restrained occupants (Figure 2). Unrestrained front- and unrestrained rear-seated occupants who were transferred to another acute care facility incurred medical charges that were more than double similarly seated restrained occupants (Figure 2). Unrestrained front-seated male occupants had median charges that were more than double those of similarly seated restrained male occupants. Among the severely injured who did not die, hospital charges were more than three-fold higher for unrestrained occupants. Among occupants traveling in cars or light trucks, the disparity in medical charges for unrestrained compared to restrained occupants was greater in front- and rear-seated occupants (Table S1, Supplementary Materials). More than 95% of ejections were unrestrained and encountered significantly increased median medical charges (not shown).

*Median charges in alcohol-involved and speed-related crashes.* For alcohol-involved crashes, medical charges were nearly doubled in front-seated unrestrained occupants compared to front-seated restrained occupants (not shown in table). For speed-related crashes, charges were higher in both front-and rear-seated occupants regardless of restraint status. Unrestrained front-seated occupants in speed-related crashes had charges that were nearly four-fold higher than front-seated restrained occupants not involved in a speed-related crash. Similarly, for rear-seated occupants charges were more than double for unrestrained occupants involved in a speed-related crash.

*Source of payment for medical charges.* Medical charges included all charges incurred during a visit to an emergency department or during hospitalization. Approximately one-fourth of occupants were covered by governmental insurance or were uninsured/self-insured, but this tended to be higher in unbelted than in restrained occupants who were front-seated (28.3% vs. 23.7%, *p* < 0.00001, not shown) and who were rear-seated passengers (27.4% vs. 25.4%, *p* = 0.0043, not shown in table).

## 4. Discussion

The findings of this study extend and update the science of adult rear-seat motor vehicle occupant injury and associated costs related to restraint use and non-use and place it in the context of similarly restrained and unrestrained front-seated occupants. Key findings of this examination of injury and hospital charges in restrained and unrestrained front- and rear-seated adults are that: (1) rear-seated adults were one-fifth as likely to be restrained as front-seated occupants; (2) among the rear-seated, unbelted severe injury and death was triple and lengths of hospitalization stay were nearly double that of restrained rear-seated passengers; (3) total length of hospitalization stay tended to be longer in rear-seated than front-seated passengers with the difference being driven by the substantially higher proportion of unrestrained rear-seated passengers; (4) significant societal financial burden is evidenced as more than one-quarter of unrestrained occupants in both the front- and rear-seating locations were covered by public insurance/self-pay or insured; (5) among restrained occupants, rear-seated median length of hospitalization stay was one day shorter than for front-seated occupants.

Our study updates and extends earlier studies that also note increased hospitalization and emergency department charges associated with being unrestrained compared to restrained, although many of these studies are very informative for the pediatric age range or for mixed age populations [31,32,33,34,35]. By limiting the current study population to adults aged 18 and older, it was possible to examine charges and costs specific to the adult rear-seated occupant and to investigate differential injury and charges by restraint status in adult rear- vs. front-seating positions. Although vulnerable road users, such as pedestrians and children are also of importance, this particular study focused on adult motor vehicle occupants. The latter is important because of the paucity of information available to inform policy makers and legislators many of whom are debating what the best practices should be for rear-seat restraint laws in their state. Much of the historical work on rear-seat injury costs excludes adults, includes both adults and children or is decades old—Which falls short of providing evidence to inform extending seat belt legislation coverage to adults [32,33,34,35,36,37].

A strength of this study is the use of the CODES data base which includes nonfatal and fatal occupants, vehicle and crash characteristics merged with hospitalization and emergency department visits, including charge data [21,22,23]. Although many of the factors associated with restraint use in front- and rear-seated occupants are similar to earlier findings using the Fatality Analysis Reporting System, the current study using the CODES database includes nonfatal crashes and treatment charge information which is more representative of all injurious motor vehicle crashes [21,24].

A novelty of this study it that it examines outcomes and charges by seating position specific to adult occupants. This study also provides an update to the injury outcomes work performed two and three decades ago. The finding of higher restraint use in females, older ages, weekday crashes, daytime crashes, and being unimpaired is consistent with prior studies [1,12,14,15,31]. This study is also consistent with previous studies, performed in children or that included child occupants, that found reduced injury severity and/or lower hospital charges in restrained occupants [2,11,18,31,32,33,34,35].

This study’s finding of higher charges/costs associated with being unrestrained is consistent with previous work that found 25–186% increased charges in unrestrained versus restrained motor vehicle occupants [31,32,33,34]. Our finding of increased injury and charges in rear-seated adult passengers is driven by the increased incidence of being unrestrained at the time of crash. Many previous studies were completed two and three decades ago prior to changes in seat belt use, before the widespread improvements in the occupant cage of front-seated drivers and before the introduction of rear-seat compartment improvements such as side curtain and rear-seat airbags [17,27]. Many new vehicle improvements have been introduced, initially for the front seat, and were not widely available in the rear seat of the vehicle fleet analyzed for this study. Given the long lead time until there is fleet saturation of new safety technology, more immediate gains could be realized by narrowing the gap in seat belt use between the front- and rear-seated occupants [17]. Driver characteristics, such as driver education training, were not available for analysis in the CODES data set.

This study had limitations. It is important to note that hospital charges do not represent the total cost of the motor vehicle crash. This study, as many previous studies, operationalized costs associated with injury as hospitalization and emergency department charges, and likely underestimates the true charges of injury which include physicians’/professional fees, rehabilitation, or follow-up care [12,24,31,33,36]. This study may have missed charges for minor injury where the injured person did not seek or receive medical treatment at a hospital, or where a crash in NYS occurred in a border county with treatment being delivered out of state. It was not feasible to assess charges associated with being unrestrained where restraint status was missing for 11.2% of front-seated occupants and for 13.7% of rear-seated occupants. Any errors in identifiable fields used for data linkage may result in a failure to link cases to their crash record. It is possible for reporting errors to be made by the motor vehicle occupant, public safety personnel, data transcribers or other health professionals. However, there is little reason to believe that this occurred differentially between restrained and unrestrained or between front- and rear-seated occupants. This study focuses only on motor vehicle occupants and does not include the costs of motor vehicle crashes incurred by vulnerable road users, such as pedestrians.

Many historical studies did not report seating position and only included hospital charges and not professional fees or rehabilitation. There is some variability in indicators and cost estimates across studies. Earlier work noted that charges differed by 25% to 64% for restrained and unrestrained occupants for injuries treated in all hospitals, with one study noting an 87% restraint use differential for injuries treated in trauma centers, possibly reflecting a difference in case mix [11].

## 5. Conclusions

In summary, this study challenges the widespread fallacy that adult passengers do not need to be restrained to travel safely in the rear seat and supports use of effective counter measures for improving adult rear-seat safety. It documents disparities in adult restraint use by seating position. Compared to restrained adults, lack of restraint use during a motor vehicle collision was associated with higher serious injury and medical charges in both front- and rear-seated adults. It is important to note that at the time of this study, there was no rear-seat belt law in NYS covering persons aged 16 and older unless they were being driven by a junior licensed driver. Lack of restraint use in adult rear-seated motor vehicle occupants consumes scarce health care dollars for treatment of this serious, but largely preventable injury. Further study is needed to assess factors related to the enforcement and impact of NYS’s restraint laws that were strengthened during the pandemic and now require all ages in all seating positions to be restrained.

## Figures and Tables

**Table 1:** Population Characteristics for Adult Occupants Involved in a Motor Vehicle Crash in New York State, Front-Seated and Rear-Seated Occupants by Restraint Status, Crash Outcome Data Evaluation System (CODES), 2016–2017.

	Front-Seated Occupants (*n*,%)		Rear-Seated Occupants (*n*,%)		Total (*n*,%)		*X*^2^ Front vs. Rear
	Restrained	Unrestrained	Restrained	Unrestrained	Front-Seated ^1^	Rear-Seated ^1^	*X*^2^ (*p*-Value)
**Study population (Total *N*)**	110,843	5096	4515	1214	130,671	6641	
**Driver characteristics**							
**Driver age (in years)**			NA ^2^		NA ^2^		
≤25	20,055 (22.9)	715 (26.3)					
26–44	35,244 (40.3)	1171 (43.1)					
45–64	28,220 (32.3)	732 (26.9)					
65+	3955 (4.5)	100 (3.7)					
**Driver sex/gender**			NA^2^		NA^2^		
Male	49,580 (52.7)	2079 (72.3)					
Female	44,430 (47.3)	796 (27.7)					
**Passenger characteristics**							
**Passenger age (in years)**							<0.0001
≤25	4312 (24.5)	169 (32.5)	1446 (32.0)	364 (30.1)	4474 (24.9)	2044 (16.1)	
25–44	6196 (37.6)	228 (43.8)	1689 (37.4)	525 (43.4)	6837 (37.0)	2595 (37.1)	
45–64	4101 (24.9)	85 (16.3)	958 (21.2)	247 (20.4)	4404 (24.5)	1425 (21.5)	
65+	2160 (13.1)	38 (7.3)	422 (9.3)	73 (6.0)	2289 (12.7)	567 (25.7)	
**Passenger sex/gender**							0.1258
Male	6022 (36.5)	258 (49.6)	1713 (38.0)	517 (42.8)	6702 (37.3)	2538 (38.3)	
Female	10,465 (63.5)	262 (50.4)	2796 (62.0)	691 (57.2)	11,290 (62.8)	4086 (61.7)	
**Injury severity and health care characteristics**							
**Disposition of inpatients**							0.692
Home, no care	106,630 (96.2)	4616 (90.6)	4353 (96.4)	1153 (95.0)	124,975 (95.6)	6359 (95.8)	
Home, home health care	427 (0.39)	65 (1.3)	15 (0.33)	7 (0.58)	673 (0.52)	35 (0.53)	
Other acute care facility	1742 (1.5)	209 (4.1)	58 (1.3)	29 (2.4)	2388 (1.8)	119 (1.8)	
Skilled nursing facility	373 (0.34)	47 (0.92)	8 (0.18)	4 (0.33)	504 (0.39)	17 (0.26)	
Died	219 (0.20)	59 (1.2)	6 (0.13)	9 (0.74)	372 (0.28)	18 (0.27)	
Other	1452 (1.3)	100 (2.0))	75 (1.7)	12 (0.99)	1759 (1.4)	93 (1.4)	
**Injury severity**							<0.0001
Uninjured	49,609 (44.8)	733 (14.4)	1902 (42.1)	271 (22.3)	55,972 (42.8)	2412 (36.3)	
Minor	49,632 (44.8)	2434 (47.8)	2192 (48.6)	665 (54.8)	57,947 (44.4)	3312 (49.9)	
Moderate	5783 (5.2)	983 (19.3)	218 (4.8)	146 (12.0)	8239 (6.3)	482 (7.3)	
Severe	4153 (3.8)	762 (15.0)	120 (2.7)	106 (8.7)	6244 (4.8)	293 (4.4)	
Killed	137 (0.12)	81 (1.6)	1 (0.02)	9 (0.74)	320 (0.24)	13 (0.20)	
Unknown severity	1529 (1.4)	103 (2.0)	82 (1.8)	17 (1.4)	1949 (1.5)	129 (1.9)	
**Length of stay (in days) ^3^**							
Mean, SD	5.19, 7.38	7.02, 9.50	4.17, 5.07	7.55, 10.52	5.87, 8.31	6.19, 8.25	0.18
**Payment Characteristics**							
**Source of payment**							<0.0001
Private	70,229 (65.8)	2963 (60.3)	2763 (63.5)	713 (60.6)	82,148 (65.2)	3999 (62.5)	
Governmental source	11,012 (10.3)	543 (11.1)	499 (11.5)	136 (11.6)	13,196 (10.5)	744 (11.6)	
Workers’ compensation	5588(5.2)	240 (4.9)	210 (4.8)	62 (5.3)	6427 (5.1)	322 (5.0)	
Self-pay/uninsured	14,325 (13.4)	846 (17.2)	607 (14.0)	187 (15.9)	17,354 (13.8)	938 (14.7)	
Other	2260 (2.1)	84 (1.7)	88 (2.0)	26 (2.2)	2629 (2.1)	123 (1.9)	
Unknown	3381 (3.2)	235 (4.8)	183 (4.2)	53 (4.5)	4201 (3.3)	276 (4.3)	

^1^ Includes occupants with unknown restraint status. ^2^ NA, Not applicable ^3^ Data were skewed.

**Table 2:** Vehicle and Crash Characteristics for Adult Occupants Involved in a Motor Vehicle Crash in New York State, Front-Seated and Rear-Seated Occupants by Restraint Status, Crash Outcome Data Evaluation System (CODES), 2016–2017.

	Front-Seated Occupants (*n*,%)		Rear-Seated Occupants (*n*,%)		Total (*n*,%)		*X*^2^ Front vs. Rear
	Restrained	Unrestrained	Restrained	Unrestrained	Front-Seated ^1^	Rear-Seated ^1^	*X*^2^ (*p*-Value)
**Study population (*N*)**	110,843	5096	4515	1214	130,671	6641	
**Vehicle characteristics**							
**Vehicle body type**							<0.0001
Car	58,971 (54.2)	1734 (37.5)	2334 (52.4)	758 (64.4)	65,932 (53.2)	3553 (55.9)	
Light Truck	48,244 (44.3)	1213 (26.3)	2109 (47.3)	416 (35.3)	53,573 (43.1)	2776 (43.7)	
Large truck	1586 (1.5)	48 (1.0)	14 (0.31)	1 (0.08)	1798 (1.5)	16 (0.25)	
Other	1926 (1.7)	151 (7.3)	56 (71.8)	22 (28.2)	3051 (2.5)	10 (0.16)	
**Vehicle year**							<0.0001
<1994	7435 (6.7)	1863 (36.6)	377 (8.4)	101 (8.3)	12,743 (9.8)	595 (9.0)	
1994–1997	2597 (2.3)	96 (1.9)	61 (1.4)	21 (1.7)	2977 (2.3)	97 (1.5)	
1998–2004	21,703 (19.6)	750 (14.7)	753 (16.7)	200 (16.5)	24,786 (19.0)	1102 (16.6)	
2005–2008	22,017 (19.9)	684 (13.4)	842 (18.7)	254 (20.9)	25,115 (19.2)	1249 (18.8)	
2009–2011	9577 (8.6)	244 (4.8)	363 (8.0)	98 (8.1)	10,790 (8.3)	529 (8.0)	
2012 or newer	47,492 (42.9)	1246 (24.5)	2118 (46.9)	540 (44.5)	53,913 (41.3)	3068 (9.0)	
Unknown	22 (0.02)	213 (4.2)	1 (0.02)	0 (0)	347 (0.27)	1 (0.02)	
**Crash characteristics**							
**Ejection**							<0.0001
Not ejected	109,076 (98.4)	3798 (74.5)	4459 (98.8)	1148 (94.6)	123,214 (94.3)	6350 (95.6)	
Partially ejected	87 (0.08)	219 (4.3)	6 (0.13)	9 (0.7)	734 (0.6)	37 (0.6)	
Ejected	52 (0.05)	958 (18.8)	2 (0.04)	43 (3.5)	3201 (2.5)	172 (2.6)	
Unknown	1628 (1.5)	121 (2.4)	48 (1.)1	14 (1.2)	3522 (2.7)	82 (1.2)	
**Airbag deployment**							<0.0001
Yes	13,514 (12.2)	1177 (23.1)	144 (3.2)	37 (3.1)	14,697 (11.3)	183 (2.8)	
No	96,996 (87.5)	1919 (37.7)	4371 (96.8)	1160 (95.6)	98,936 (75.7)	5561 (83.7)	
Unknown	333 (0.30)	2000 (39.3)	0 (0)	17 (1.4)	17,038 (13.0)	897 (13.5)	
**Vehicle towed**							<0.0001
Yes	56,009 (50.5)	2275 (44.6)	2015 (44.6)	594 (48.9)	62,668 (48.0)	3014 (45.4)	
No	54,834 (49.5)	1197 (23.5)	2500 (55.4)	615 (50.7)	65,952 (49.7)	3617 (54.5)	
Unknown	0 (0)	1624 (31.9)	0 (0)	5 (0.4)	3051 (2.3)	10 (0.15)	
**Alcohol involved crash**							<0.0001
Yes	1670 (1.5)	342 (6.7)	NA ^2^	NA ^2^	2369 (1.8)	0 (0)	
No	78,888 (71.2)	3326 (65.3)			89,792 (68.7)	6 (0.09)	
Unknown	30,285 (27.3)	1428 (28.0)			38,510 (29.5)	6635 (99.9)	
**Speed involvement**							0.0021
Yes	7808 (7.0)	619 (12.2)	303 (6.7)	150 (12.4)	9431 (7.2)	546 (8.2)	
No	103,035 (93.0)	4477 (87.9)	4212 (93.3)	1064 (87.6)	121,240 (92.8)	6095 (91.8)	
**Location**							<0.0001
Urban	29,945 (27.2)	1061 (20.9)	1005 (22.3)	236 (19.5)	33,989 (26.2)	5232 (79.0)	
Rural	80,261 (72.8)	4020 (79.1)	3496 (77.7)	972 (80.5)	95,888 (73.8)	1389 (21.0)	
**Evening crash**							0.2365
Yes	29,867 (26.9)	1306 (25.6)	1310 (81.7)	293 (18.3)	34,930 (26.7)	1819 (27.4)	
No	80,976 (74.4)	3790 (74.4)	3205 (77.7)	921 (22.3)	95,741 (73.3)	4822 (72.6)	

^1^ Includes occupants with unknown restraint status. ^2^ NA Data not reported for rear-seated occupants.

## Data Availability

This is state data that is linked under a data use agreement and an IRB that does not provide for public data distribution.

## Supplementary Materials

The following supporting information can be downloaded at: https://www.mdpi.com/article/10.3390/ijerph192013674/s1, Table S1: Median Hospital Charge for Front-Seated and Rear-Seated Occupants by Restraint Status, and Total Charges for Front and Rear-Seated Occupants, Crash Outcome Data Evaluation System, 2016–2017.
