# Supplementary material for: Motor Vehicle Crash and Hospital Charges in Front- and Rear-Seated Restrained and Unrestrained Adult Motor Vehicle Occupants
Source: Int J Environ Res Public Health. 2022 Oct 21;19(20):13674. doi: 10.3390/ijerph192013674 (PMC9603584; doi:10.3390/ijerph192013674)
Supplement: Supplementary file 1 [file ijerph-19-13674-s001.zip › ijerph-1960426-supplementary.pdf]

Table S1. Median Hospital Charge for Front-Seated and Rear-Seated Occupants by Restraint Status, and Total Charges for Front and Rear-Seated Occupants, Crash Outcome Data Evaluation System, 2016-2017.

	Front-Seated Occupants (median, range in $)		*Rear-Seated Occupants (median, range in $)*		*Total (median, range in $)*	
	Restrained	*Unrestrained*	*Restrained*	*Unrestrained*	*All Front-Seated*	*All Rear-Seated*
**Study population**						
Occupant characteristics						
**Age**						
18-20	2,301 (1,185, 4,200)	3,929 (1,669, 12,058)	1,955 (1,133, 3,828)	3,171 (1,404, 8,826)	2,105 (1,206, 4,395)	2,146 (1,201, 4,917)
21-24	2,068 (1,283, 4,210)	4,350 (1,909, 12,254)	1,957 (1,236, 3,636)	2,796 (1,344, 6,993)	2,150 (1,305, 4,443)	2,131 (1,312, 4,371)
25-34	2,167 (1,302, 4,399)	3,355 (1,741, 10,347)	1,993 (1,235, 3,731)	2,298 (1,341, 5,854)	2,193 (1,312, 4,564)	2,104 (1,267, 4,197)
35-44	2,257 (1,377, 4,364)	3,571 (1,501, 10,820)	2,069 (1,253, 3,829)	2,363 (1,341, 5,285)	2,264 (1,376, 4,476)	2,150 (1,291, 4,209)
45-54	2,279 (1,423, 4,560)	3,245 (1,601, 16,449)	2,262 (1,297, 4,240)	2,240 (1,366, 5,746)	2,292 (1,413, 4,628)	2,317 (1,328, 4,801)
55-64	2,453 (1,434, 5,165)	3,920 (1,657, 5,684)	2,385 (1,328, 4,620)	2,549 (1,497, 5,816)	2,479 (1,423, 5,142)	2,462 (1,401, 5,247)
65-74	3,075 (1,674, 6,701)	14,940 (4,489, 45,998)	2,416 (1,375, 5,635)	3,870 (1,658, 8,195)	3,090 (1,684, 6,878)	2,812 (1,388, 5,839)
75-84	3,335 (1,751, 8,567)	5,750 (2,062, 10,352)	3,636 (2,190, 7,166)	2,723 (2,190, 5,870)	3,353 (1,741, 8,643)	3,513 (2,153, 7,134)
85+	4,482 (2,181, 13,307)	2,883 (2,262, 7,030)	4,804 (1,950, 11,525)	4,991 (1,828, 26,537)	4,386 (2,145, 13,195)	4,729 (1,950, 11,525)
**Sex/Gender**						
Male	2,316 (1,377, 4,758)	4,736 (1,941, 16,578)	3,920 (1,741, 13,255)	2,636 (1,358, 8,167)	2,366 (1,387, 5,091)	2,198 (1,287, 4,953)
Female	2,313 (1,376, 4,871)	3,493 (1,740, 9,968)	3,647 (1,679, 9,565)	2,465 (1,397, 5,544)	2,338 (1,379, 4,841)	2,247 (1,323, 4,651)
Injury severity and health care characteristics						
**Disposition of admitted patients**						
Home, no care	2,304 (1,394, 4,524)	3,242 (1,675, 8,432)	2,105 (1,262, 3,991)	2,378 (1,344, 5,477)	2,366 (1,419, 4,737)	2,181 (1,295, 4,366)
Home, home health care	51,337 (28,663, 164,019)	72,749 (42,417, 128,661)	40,844 (13,623, 91,752)	62,775 (26,326, 186,770)	57,653 (32,650, 102,564)	44,578 (23,252, 108,086)
Other acute care facility	11,440 (4,594, 34,475)	26,672 (9,066, 119,985)	12,350 (4,421, 43,821)	26,864 (7,557, 157,479)	14,162 (5,371, 59,046)	14,855 (5,775, 103,843)

Skilled Nursing	74,344 (28,465, 147,116)	110,920 (59,912, 228,872)	45,951 (31,005, 89,818)	79,042 (49,160, 182,848)	83,980 (42,294, 167,475)	62,995 (37,617, 119,310)
Died	18,157 (4,241, 83,547)	14,426 (3,394, 68,812)	49,245 (15,987, 185,644)	14,309 (5,014, 48,996)	16,439 (4,236, 77,033)	19,065 (6,457, 64,924)
Other	2,537 (1,196, 7,506)	6,976 (1,865, 18,513)	1,657 (870, 4,274)	2,211 (847, 7,379)	2,816 (1,257, 9,230)	1,701 (870, 4,551)
**Injury severity**						
Uninjured	2,007 (1,245, 3,666)	2,272 (1,333, 4,704)	1,866 (1,176, 3,311)	1,870 (1,201, 3,259)	2,016 (1,256, 3,709)	1,877 (1,188, 3,380)
Minor	2,409 (1,453, 4,745)	2,974 (1,602, 7,269)	2,153 (1,277, 4,101)	2,110 (1,231, 4,505)	2,434 (1,467, 4,895)	2,169 (1,278, 4,278)
Moderate	3,090 (1,584, 7,465)	3,895 (1,888, 10,043)	2,941 (1,676, 6,174)	3,663 (1,822, 7,826)	3,332 (1,663, 8,467)	3,110 (1,715, 6,751)
Severe	5,538 (2,164, 18,976)	18,282 (5,154, 54,894)	4,421 (1,907, 13,361)	19,935 (4,928, 51,899)	7,840 (2,601, 29,959)	10,304 (2,596, 35,464)
Killed	14,044 (3,594, 76,036)	11,186 (3,017, 48,320)	49,245 (49,245, 149,423)	10,363 (3,788, 36,620)	12,513 (3,292, 65,580)	14,309 (6,349, 49,121)
Unknown Severity	2,053 (1,287, 4,033)	2,847 (1,587, 7,525)	1,638 (881, 3,146)	2,495 (1,644, 4,035)	2,110 (1,299, 4,322)	1,741 (1,071, 3,272)
Vehicle characteristics						
**Vehicle body type**						
Car	2,383 (1,429, 4,839)	3,737 (1,741, 12,010)	2,145 (1,270, 4,154)	2,433 (1,338, 5,600)	2,389 (1,433, 4,905)	2,205 (1,308, 4,531)
Light Truck	2,378 (1,401, 4,768)	4,270 (1,811, 13,103)	2,140 (1,264, 4,203)	2,673 (1,418, 7,792)	2,390 (1,410, 4,867)	2,184 (1,290, 4,632)
Large truck	2,485 (1,438, 6,158)	4,647 (2,151, 14,903)	2,948 (1,873, 4,675)	2,916 (2,916, 2,916)	2,599 (1,478, 6,475)	2,916 (2,149, 3,759)
Other	2,053 (1,287, 4,033)	3,053 (1,701, 7,581)		2,042 (1,164, 9,646)	3,069 (1,695, 7,536)	2,879 (1,514, 4,982)
**Vehicle year**						
<1994	2,195 (1,336, 4,452)	3,125 (1,674, 8,370)	2,008 (1,240, 3,659)	2,100 (1,235, 4,956)	2,488 (1,447, 5,593)	2,094 (1,258, 4,068)
1994-1997	2,641 (1,516, 5,863)	7,242 (3,001, 21,539)	2.420 (1,445, 7,184)	3,321 (1,577, 10,074)	2,831 (1,562, 6,709)	2,613 (1,505, 6,742)
1998-2004	2,459 (1,448, 5,205)	4,958 (2,180, 17,556)	2,226 (1,267, 4,503)	3,180 (1,458, 10,454)	2,548 (1,475, 5,615)	2,407 (1,344, 5,308)
2005-2008	2,332 (1,375, 4,773)	4,447 (1,814, 13,734)	2,237 (1,308, 4,255)	3,005 (1,673, 7,379)	2,411 (1,394, 5,140)	2,394 (1,400, 5,304)
2009-2011	2,354 (1,389, 4,694)	4,122 (1,803, 11,763)	2,205 (1,275, 4,479)	2,790 (1,411, 6,180)	2,400 (1,406, 4,931)	2,363 (1,365, 5,476)
2012 or newer	2,364 (1,434, 4,682)	3,491 (1,741, 11,130)	2,080 (1,238, 3,940)	2,209 (1,259, 5,073)	2,406 (1,449, 4,892)	2,143 (1,257, 4,267)
Unknown	1,691 (1,399, 3,430)	3,793 (1,985, 12,013)	1,980 (1,649, 2,312)	1,480 (1,480, 1,480)	3,745 (1,913, 13,182)	1,649 (1,649, 2,312)
Crash characteristics						
**Ejection**						
Not ejected	1,916 (1,271, 3,522)	3,365 (1,680, 9,457)	2,131 (1,244, 4,163)	2,426 (1,365, 5,746)	2,407 (1,433, 4,957)	2,184 (1,295, 4,486)
Partially ejected	3,300 (1,534, 9,905)	4,074 (1,842, 14,351)	5,763 (1,299, 12,823)	5,244 (2,331, 21,521)	4,735 (2,002, 15,974)	3,712 (2,331, 12,411)

Ejected	6,877 (2,688, 18,978)	5,760 (2,279, 20,374)	1,933 (1,756, 1,933)	14,016 (6,410, 68,776)	7,698 (2,626, 26,047)	9,765 (2,809, 35, 723)
Unknown	2,380 (1,417, 4,818)	3,000 (1,669, 12,653)	2,345 (1,240, 3,669)	1,741 (1,126, 3,863)	2,065 (1,306, 3,956)	2,345 (1,239, 4,089)
**Airbag deployment**						
Yes	3,156 (1,641, 8,308)	4,196 (1,953, 13,241)	2,623 (1,394, 7,295)	4,588 (1,394, 14,278)	3,222 (1,663, 8,602)	2,769 (1,391, 8,194)
No	2,271 (1,385, 4,449)	3,895 (1,675, 12,723)	2,116 (1,262, 4,069)	2,495 (1,380, 6,124)	2,291 (1,388, 4,536)	2,186 (1,288, 4,411)
Unknown	1,900 (1,116, 3,687)	3,322 (1,755, 9,341)	NA	2,466 (1,540, 9,646)	2,904 (1,584, 7,940)	2,509 (1,428, 6,464)
**Alcohol involvement**						
Yes	4,677 (1,910, 12,245)	9,129 (3,428, 25,673)	2,623 (1,394, 7,295)	2,466 (1,540, 9,646)	5,383 (2,160, 14,998)	NA
No	2,433 (1,449, 4,972)	3,733 (1,811, 11,859)	2,116 (1,262, 4,069)	1,949 (1,164, 3,730)	2,549 (1,489, 5,442)	2,097 (1,164, 3,633)
Uknown	2,136 (1,322, 4,181)	3,001 (1,559, 7,869)	2,138 (1,265, 4,148)	2,534 (1,382, 6,363)	2,173 (1,336, 4,324)	2,232 (1,312, 4,771)
**Speed involvement**						
Yes	2,998 (1,484, 7,664)	8,386 (2,603, 24,909)	2,575 (1,306, 6,059)	4,587 (1,819, 15,362)	3,447 (1,601, 10,173)	3,098 (1,505, 9,783)
No	2,337 (1,409, 4,647)	3,400 (1,718, 9,746)	2,106 (1,263, 4,035)	2,398 (1,341, 5,592)	2,401 (1,434, 4.945)	2,186 (1,299, 4,462)
**Payment Characteristics**						
**Source of payment**						
Private	2,380 (1,405, 4,885)	3,878 (1,833, 12,356)	2,163 (1,243, 4,364)	2,728 (1,401, 6,993)	2,456 (1,434, 5,252)	2,259 (1,308, 5,175)
Governmental source	3,020 (1,616, 7,187)	5,798 (2,047, 24,925)	2,348 (1,318, 4,606)	2,461 (1,400, 6,573)	3,160 (1,661, 8,820)	2,407 (1,355, 5,541)
Workers Compensation	2,017 (1,323, 3,591)	2,485 (1,434, 5,994)	2,105 (1,376, 3,482)	1,867 (1,200, 3,147)	2,046 (1,335, 3,676)	1,987 (1,314, 3,287)
Self-pay/uninsured	2,270 (1,370, 4,299)	3,144 (1,672, 7,705)	2,068 (1,268, 3,798)	2,398 (1,251, 5,749)	2,361 (1,393, 4,648)	2,203 (1,300, 4,310)
Other	2,247 (1,320, 4,437)	4,075 (1,809, 9,688)	2,013 (1,058, 3,305)	2,186 (1,660, 6,593)	2,298 (1,340, 4,607)	2,106 (1,163, 3,938)
Unknown	2,171 (1,601, 3,918)	3,086 (1,764, 6,584)	2,034 (1,555,3,540)	2,363 (1,475, 4,949)	2,239 (1,622, 4,160)	2,088 (1,500, 3,891)
